# Mixed Glioneural Tumors Mimicking Gliomas: Two Cases From a Tertiary Care Institute in South India and a Literature Review

**DOI:** 10.7759/cureus.78079

**Published:** 2025-01-27

**Authors:** Narendhar Gokulanathan, Prashanth Gokulanathan, Nirmal Bharathi, P Subburam

**Affiliations:** 1 Medical Oncology, Apollo Hospitals, Bangalore, IND; 2 Anesthesiology and Critical Care, Melmaruvathur Adhiparasakthi Institute of Medical Sciences, Melmaruvathur, IND; 3 Critical Care Medicine, Orthomed Hospital, Chennai, IND; 4 Neurological Surgery, Mahatma Brain and Spine Centre, Madurai, IND

**Keywords:** brain tumors, chemotherapy, epilepsy, gliomas, glioneuromas, neurooncology, neurosurgery, oncology, radiation, radiotherapy

## Abstract

Glioneuronal tumours are rare neural neoplasms that show differentiation and are usually low-grade. Unlike gliomas, they do not require aggressive management, and diagnosing and differentiating between these two entities is crucial to determining the treatment paradigm of this disease. Surgery is the treatment of choice for glioneuronal tumours, and the role of adjuvant treatment still needs to be fleshed out. These two cases, involving a six-year-old female child and a 35-year-old male patient, highlight the need to be cautious while differentiating these entities from their equivalents with poorer prognoses. They both underwent surgery followed by adjuvant radiation, with subsequent neuroimaging at regular intervals. Both patients were devoid of neurological complaints at the time of follow-up. These cases and literature review delve into the minutiae of clinical manifestations, histopathological characteristics, various modalities of treatment, and treatment outcomes of glioneuromas.

## Introduction

Glioneuronal tumours are rare, predominantly low-grade neural neoplasms that show differentiation. Accurate diagnosis and differentiation between glioneuronal tumors and gliomas are crucial, as this distinction directly influences the treatment approach and outcomes. Surgical resection remains the cornerstone of treatment for glioneuronal tumors, although the precise role of adjuvant therapies such as radiation and chemotherapy remains under investigation [1-3].

This case series highlights two patients--a young child and an adult male--who underwent surgical intervention followed by adjuvant radiation therapy, emphasizing the importance of careful histopathological and molecular assessment to distinguish these tumors from their more aggressive counterparts.

## Case presentation

Case 1

In February 2021, an apparently asymptomatic six-year-old girl developed multiple episodes of generalized tonic-clonic seizures for a week and visited our hospital. She did not have any other features of raised intracranial pressure or any other features suggestive of central and peripheral nervous system involvement. Magnetic resonance imaging of the brain showed a well-defined isotense to a hyperintense lesion in the right temporal region involving the hippocampus and parahippocampal gyrus, measuring 38x32x17 mm. The lesion did not show diffusion restriction, increased perfusion, post-contrast enhancement, or lipid lactate peak. Magnetic resonance spectroscopy showed focal areas of elevated choline levels, suggestive of a low-grade glioma or a dysembryoplastic neuro-epithelial tumour. She underwent right temporal craniectomy and decompression of lesion with middle and inferior temporal gyrus corticectomy on 16th March 2021. Watertight dural closure was done using the pericranium, and the bone flap was replaced and fixed with an absorbable Craniofix (manufactured by B-Braun). Her post-operative period was uneventful.

**Figure 1:**
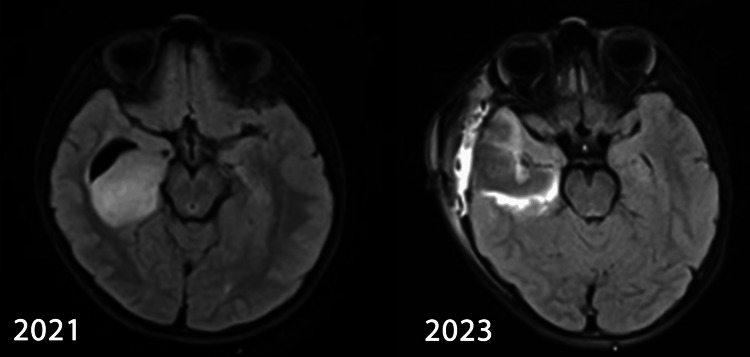
MRI of Patient 1 at baseline and most recent follow up

Histopathological examination showed a temporal cortical-based tumour. The tumour was low to moderately cellular and composed of oligodendroglia-like cells with a distinct round nucleus and perinuclear clearing, loosely spaced in the cortex with a few entrapped floating neurons in an eosinophilic matrix, suggestive of glioneuroma. A prominent and delicate vasculature pattern was noted. No atypical ganglioid cells were noted. Areas of fresh haemorrhages were noted within the tumour. Mitotic activity was inconspicuous, and the interstitium was delicately fibrillar.

Immunohistochemistry showed IDH 1 and P53 negative, in accordance with glioneuromas. ATRX was retained. CD34 highlighted the vasculature only. Synaptophysin highlighted the cortical and floating neurons. MIB-1 labelling index was 2-3%. Overall histological features were compatible with low-grade glioneuronal tumours, favouring a dysembryoplastic neuroepithelial tumour of the right medial temporal region, WHO Grade 1.

Post-op MRI was suggestive of a residual lesion. Due to the young age of the patient and the imminent long-term dangers of cranial irradiation in said age group, such as neurocognitive deficits and second malignancies, she underwent repeat excision of the tumour to achieve gross total resection. After counselling the child’s parents accordingly, adjuvant radiotherapy was not considered as gross total resection was achieved. As of October 2023, the child was healthy and had no neurological deficits or seizure episodes. Review neuroimaging did not show any change in the primary lesion. Her academic performance was not affected.

Case 2

A 35-year-old gentleman, with an established diagnosis of neurofibromatosis Type 1, presented with seizures, vomiting, and headache in 2009. He was diagnosed with a right temporoparietal space-occupying lesion and underwent craniectomy and gross total resection (GTR) in October 2009. He had an uneventful post-operative period and had no neurocognitive defects following the surgery. The post-operative histopathological examination revealed pilocytic astrocytoma, WHO Grade 1. He did not receive any adjuvant treatment. He was started on antiseizure medication, remained asymptomatic for three years, and was on regular follow-up.

In February 2013, he presented with neck pain, blurring of vision, and headache and was evaluated for the same. MRI revealed a recurrent lesion at the right temporoparietal post-operative region. He underwent re-exploration and GTR. His post-operative recovery period was uneventful.

Histopathology showed a cellular tumour originating from the right temporoparietal cortex, composed of small round neurocytic-like cells and spindle-shaped glial cells, with long processes arranged around thin and thick-walled vessels. The tumour had moderate mitotic activity and a MIB-1 labelling index of 8%. It showed cystic change, and the cyst wall was lined by tumour neovascularisation, interspersed in an eosinophilic matrix. Microvascular proliferation and necrosis were seen as well. The tumour cells and processes had diffuse synaptophysin positivity, and ganglion cells were GFAP positive, suggestive of an anaplastic ganglioglioma. 

In view of recurrence, he received adjuvant radiation treatment, to a total dose of 59.4 Grays in 33 fractions. The course of radiation and the post-treatment follow-up period was uneventful, and he did not have any neurocognitive or focal neurological defects till September 2019. 

In September 2019, he developed recurrent headaches, memory disturbances, altered sleep cycles, and decreased concentration along with nausea, and recurrence was suspected. MRI as visualised below in Figure 1 showed post-operative and post-radiation gliotic changes with increased choline and decreased n-acetyl aspartate on spectroscopy. A sub-centimetric lesion was observed near the splenium of the corpus callosum, suggestive of recurrence.

He underwent further molecular pathologic analyses. CD34 was positive in the tumour vasculature. The tumour had retained expression of ATRX, negativity for Olig-2, IDH1(R32H), EMA, BRAF V600E, and p53, which were suggestive of an anaplastic ganglioglioma of the temporoparietal region, WHO Grade 3.

**Figure 2:**
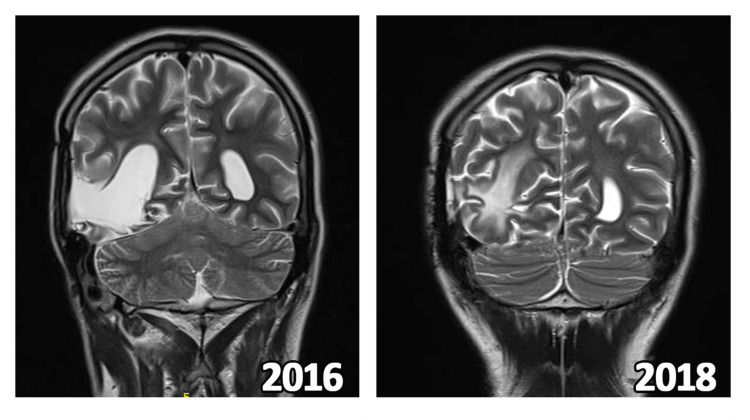
MRI of Patient 2 during various phases of follow up in 2016 and 2018

However, he could not visit the hospital from March 2020 to January 2021 because of the coronavirus pandemic. After discussion with the multidisciplinary molecular tumour board, the patient was started on palliative intent, single agent six-weekly lomustine 110 mg/m2. He completed 12 cycles in March 2022.

Response assessment MRI as shown in Figure 2 showed a nodular altered signal-enhancing lesion in the right side of the splenium of corpus callosum with elevated choline-creatinine ratio on MRS, suggestive of a stable disease.

**Figure 3:**
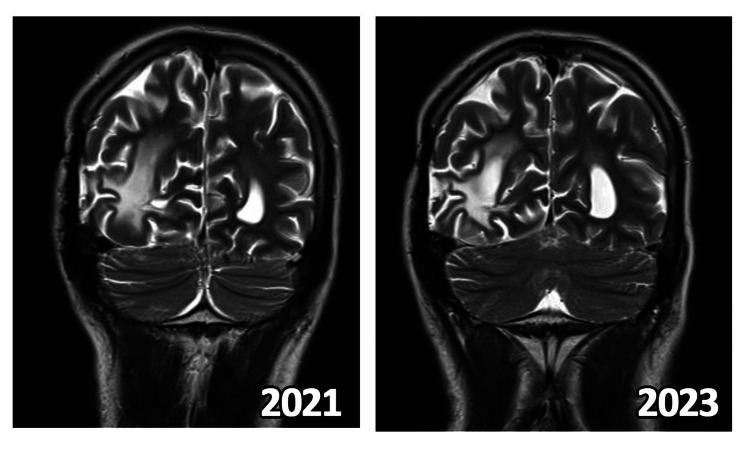
MRI of Patient 2 after initiation of lomustine and most recent imaging follow up

The patient did not experience any seizures or neurological deficits and is scheduled for follow-up with clinical evaluation and neuroimaging every six months. As of October 2023, the patient has had no seizures, neurological complaints, or deficits.

## Discussion

Glioneuronal or dysembryoplastic neuroepithelial tumours (DNETs) were first described in 1988 as a surgically curable cause of intractable partial seizures in patients aged 1-19 (mean nine years) [1]. DNETs, which are rarely seen in adults, have an incidence of 1.2% among all neuroepithelial tumours in patients under 20 years of age, with a male preponderance [2].

Their low proliferative potential and the possibility of a cure from surgical resection classified them under the indolent WHO Grade 1 tumour, along with other neuronal and mixed neuronal-glial entities like ganglioglioma, gangliocytoma, anaplastic ganglioglioma, papillary glioneuronal tumour, neurocytoma, liponeurocytomas and paragangliomas. Other inclusions into the family of glioneuronal tumours are glioneuronal tumours with neuropil-like islands (GNTNI), rosette-forming glioneuronal tumours of the fourth ventricle (RGNT), diffuse leptomeningeal glioneuronal tumour (DLGNT) and desmoplastic infantile ganglioglioma/astrocytoma (DIG/DIA) [3].

DNETs present as chronic and early onset intractable complex partial to drug-resistant generalised tonic-clonic seizures [4]. Other clinical manifestations include hydrocephalus, papilledema, blurred vision, headache and visual field defects. It also manifests as motor deficits in large lesions, with associated sensory deficits and memory loss, especially in tumours of the temporal region. Memory loss is commonly associated with post-operative status and can herald recurrence [5,6]. DNETs generally localise towards the supratentorial cortex and involve the temporal lobe (especially the hippocampus) in around 50% of the patients. Perilesional oedema, if present, is usually representative of a tumour with a marked mass effect and a higher proliferative index of >5% [7,8].

The tumours vary in appearance from well to poorly demarcated, with cyst formation, and are soft to firm in consistency. The white-grey matter line is frequently blurred but very rarely involved [1].

The most common histological subtypes of low-grade glioneuronal tumours are gangliogliomas and DNETs. They show dysplastic and neoplastic glial and neuronal tissue floating in a pale eosinophilic matrix. Glial fibrillary acidic protein (GFAP) is observed in the glial component of the tumours and synaptophysin is seen in the neuronal or ganglion cell components. The tissues that are positive for both markers form solid pseudopapillae with perivascular lymphocytic infiltrates, microcalcifications, and weakly PAS-positive eosinophilic granular bodies. Columns comprising bundles of axons are arranged perpendicular to the cortical surface. Neuronal nuclear antigen, calcineurin, tyrosine hydroxylase and CD34 are other markers found in these tumours. Necrosis and mitoses are rare or visibly absent in low-grade glioneuromas [1,2,9,10]. 

BRAF V600E mutations, which are of therapeutic importance, are present in around 30% of gangliogliomas, DNETs and DIA/DIG, with glioneuromas in the diencephalic region showing a higher rate of BRAF positivity. DNETs are notably negative for IDH, p53 and 1p/19q co-deletion mutations [1,2,9-11].

Gangliogliomas are classified into two groups based on BRAF mutation. Group 1 tumours contain more BRAF V600E mutations and present with seizures at a median of 30.2 months. They show female preponderance and are usually situated in the midline and have an aggressive clinical course and poorer response to chemoradiation. Group 2 tumours contain more FGFR mutations and present at a median of 87.5 months [9,11,12].

Cortical dysplasia, nodular enhancement and calcifications are seen on CECT. On MRI, it is hypointense on T1, hyperintense on T2, and hypo to isointense with a hyperintense rim on T2/FLAIR sequence. It does not enhance with contrast and is usually devoid of haemorrhages. On spectroscopy, choline is elevated, n-acetyl aspartate is decreased and cerebral perfusion is reduced. There is increased amino acid uptake in amino-acid positron emission tomography [2,13]. The differential diagnoses comprise low-grade gliomas and arteriovenous malformations.

In low-grade tumours, the cornerstone of treatment is surgical resection, previously performed according to epilepsy surgery protocols. It offers symptomatic relief and doubles as a debulking measure. In patients who had pharmacoresistant epilepsy as their presenting clinical feature, lesionectomy with resection of the adjacent dysplastic cortex (or lobectomy in cases with extralesional interictal activity, detected with intraoperative EEG) had excellent seizure-free outcomes (>80% with Engels Class 1 Seizure outcomes) and local control of the tumour [14].

A baseline MRI is taken for surgical planning. Gross total resection (GTR) is defined as the removal of all tumour-related T1 enhancement and T2 FLAIR abnormality, excluding post-surgical signal change. Subtotal resection (STR) is defined as the presence of residual T1 tumour enhancement or T2 FLAIR abnormalities on postoperative MRI. The average time of recurrence of a DNET after initial resection was 6.8 years. When GTR is achieved and a Grade 1 ganglioglioma is diagnosed, adjuvant therapy has no role. If only an STR is achieved, a second surgical attempt must be made, as it affects prognosis. However, the role of adjuvant radiotherapy is not defined adequately [8,15-17].

Radiotherapy has been shown to reduce the relapse rate in high-grade tumours post-STR and can be utilised as a salvage option in recurrent low-grade tumours. However, in patients treated with radiation, there is an increased potential risk of malignant transformation to gliomas, even in completely resected tumours [8,16,17].

Anaplastic ganglioglioma (WHO 3) shows higher-grade behaviour, a more aggressive clinical course and a poorer prognosis, like gliomas [18]. In addition to GTR, radiation therapy is considered for recurrent/unresectable lesions, higher-grade glioneuromas and anaplastic ganglioglioma. Radiotherapy has reasonable local control, but concerns about the long-term side effects and second malignancies have led clinicians worldwide to proceed with chemotherapy or targeted therapies initially, despite the latter modalities achieving a poorer progression-free survival. The 15-year cumulative incidence of second malignancy is 6-8%. The second malignancies usually observed were anaplastic astrocytoma, gliosarcoma, glioblastoma, and meningioma. Underlying neurocutaneous and genetic syndromes which predispose to malignancies also must be considered while assessing second malignancy risk [15,19].

The St Jude cohort guidelines recommend a total dose of 54 Grays, in 1.8 Gray per fraction, delivered over 30 fractions, using 3D-CRT or IMRT. The GTV includes the tumour’s cystic and solid components, T2/FLAIR hyperintense and T1 enhancing areas. The surgical bed and residual T2/FLAIR hyperintense and T1 enhancing areas are included in GTV for adjuvant irradiation. A margin of 0.5 cm to 1 cm is given for the CTV. A PTV margin of 0.3 cm-0.5 cm is used for photon therapy. For tumours close to the optic tracts, the total dose should be limited to 52.2 Grays to reduce toxicity. For children under the age of five, 50 Grays can be delivered as a total dose, as shown in the CNS9702 cohort [20]. Response assessment is done using RECIST criteria on post-treatment MRIs every three months for the first two years, every six months till the fifth year and yearly thereafter [15].

Systemic therapy has not been of significant benefit and is relegated to utility in tumours unsuitable for resection, re-resection, or irradiation. Case reports utilising BRAF inhibitors, temozolomide, lomustine, bevacizumab, etoposide, vinblastine, cyclophosphamide, cisplatin, and carboplatin are available [17].

There is a progression-free survival of 85-95% of patients diagnosed with low-grade glioneuronal tumours. Tumours >6cm, or located near the optic pathway or hypothalamus and patients who underwent STR were associated with increased mortality. A larger tumour size (>6 cm) was associated with an increased risk of progression. Delayed start of radiation treatment (after at least one line of systemic therapy) did not influence survival in glioneuronal tumours [15].

Median overall survival in anaplastic ganglioglioma is less than 30 months. There is a need for long-term follow-up with clinical assessment and neuroimaging with MRI in order to avoid missing recurrences, and anaplastic change and enable early treatment of the same [8].

## Conclusions

DNET and glioneuronal tumours may often be mistaken for their glioma counterparts and can mislead the clinician towards a more aggressive treatment approach. Molecular, tissue diagnosis and imaging should be correlated to ensure that overtreatment does not jeopardise the patient’s quality of life and prevent long-term adverse effects. 

Late recurrence of DNET is rare in cases of gross total resection, hence, continued follow-up and repeat neuroimaging at regular intervals should be considered in all patients. Further exploration of molecular subtyping in this rare subset of patients will allow us to practice a more precision-based treatment protocol.
